# Chronic Wasting Disease Monitoring in Italy 2017–2019: Neuropathological Findings in Cervids

**DOI:** 10.3390/pathogens11040401

**Published:** 2022-03-26

**Authors:** Letizia Tripodi, Giuseppe Ru, Fabrizio Lazzara, Lucia Caterina Florio, Cinzia Cocco, Daniela Meloni, Mazza Maria, Elena Bozzetta, Maria Gabriella Perrotta, Maria Caramelli, Cristina Casalone, Barbara Iulini

**Affiliations:** 1 Istituto Zooprofilattico Sperimentale del Piemonte, Liguria e Valle d’Aosta, Via Bologna 148, 10154 Turin, Italy; letizia.tripodi@izsto.it (L.T.); giuseppe.ru@izsto.it (G.R.); caterinalucia.florio@izsto.it (L.C.F.); cinzia.cocco@izsto.it (C.C.); daniela.meloni@izsto.it (D.M.); maria.mazza@izsto.it (M.M.); elena.bozzetta@izsto.it (E.B.); maria.caramelli@izsto.it (M.C.); cristina.casalone@izsto.it (C.C.); 2 Dipartimento Di Prevenzione, S.C. Sanità Animale, ASL 3 Genovese, Via San Giovanni Battista 48, 16154 Genoa, Italy; fabrizio.lazzara@asl3.liguria.it; 3 Ministero della Salute, Viale Giorgio Ribotta, 00144 Rome, Italy; mg.perrotta@sanita.it

**Keywords:** CEA, cervids, chronic wasting disease, encephalopathy, neuropathological lesions, prion, TSE

## Abstract

Chronic wasting disease (CWD) is a prion disease that affects cervids; it is classified under transmissible spongiform encephalopathies (TSEs). CWD is particularly contagious, making its eradication in endemic areas very difficult and creating serious problems for cervid conservation and breeding. It has recently become an emerging public health risk to be managed by health authorities. Starting in 2017, active CWD surveillance in Italy has intensified with the monitoring of wild and farmed cervids. The present study summarizes findings from a histopathological survey of the brains from wild ruminants collected via CWD monitoring between 2017 and 2019. A total of 113 brains from 62 red deer (*Cervus elaphus*) and 51 roe deer (*Capreolus capreolus*) were submitted for analysis at the National Reference Center for Animal Encephalopathies (CEA) to determine major patterns of neuropathological lesions and correlated pathogens. Brain lesions were detected in 20 animals, 10 brain samples were unsuitable for examination, and 83 presented no lesions. Neuropathological examination revealed non-suppurative encephalitis or meningoencephalitis in most cases (15/20). This brain study revealed evidence for the absence of CWD in Italy and provided a reference spectrum of neuropathological lesions for differential diagnosis in cervids.

## 1. Introduction

Chronic wasting disease (CWD) is classified under transmissible spongiform encephalopathies (TSEs), a group of neurodegenerative diseases caused by the accumulation of misfolded isoforms of normal cellular prion proteins (PrPsc) in humans and some animal species [1]. TSEs include bovine spongiform encephalopathy (BSE), classical and atypical scrapie (Nor98) in sheep and goat, transmissible mink encephalopathy (TME), feline spongiform encephalopathy (FSE), and variant Creutzfeld–Jabob disease (vCJD) in humans. CWD is a neurodegenerative disease in cervids; its prominent features are its long incubation period and chronic progressive course with fatal outcomes.

To date, zoonotic risk is associated only with BSE; a European Food Safety Authority (EFSA) opinion [2] concluded that there is no evidence for an absolute species barrier between CWD-affected cervids and humans and that evidence for an association has never been found in epidemiological studies.

**Figure 1:**
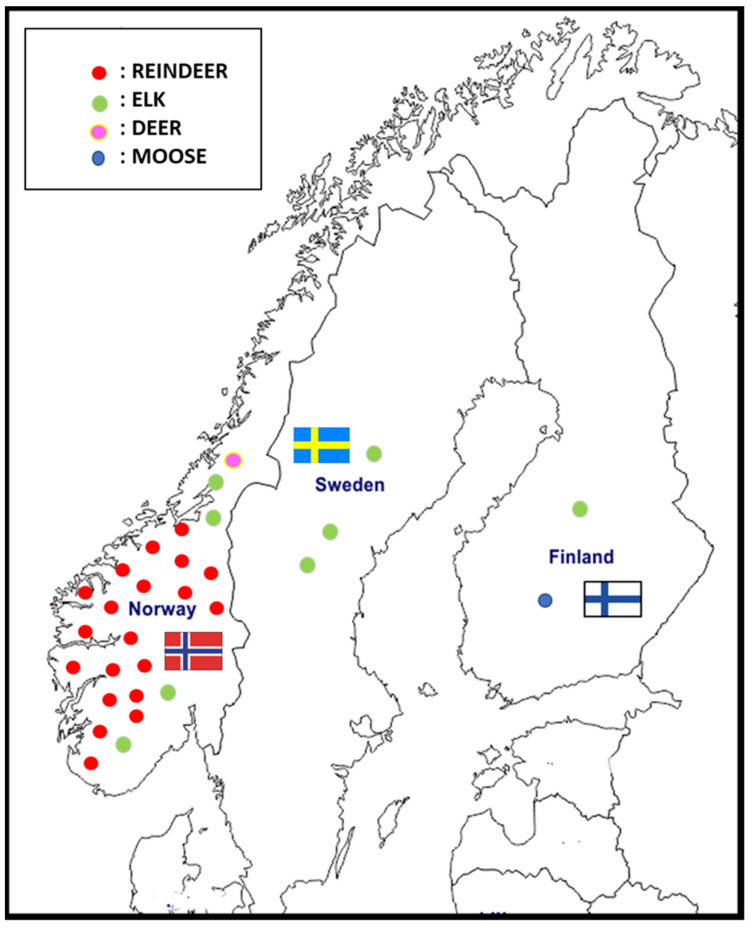
CWD cases in Norway, Sweden, and Finland reported since 2016.

The first case of CWD in Europe was reported in a free-ranging reindeer (*Rangifer tarandus*) in Nordfjella, Norway, in March 2016 [3]. Since then, 29 cases of CWD have been identified in Norway: 20 reindeer (*Rangifer tarandus*), 8 moose (*Alces alces*), and 1 red deer (*Cervus elaphus*). One of the main problems is having to manage a potential epidemic in a reindeer population characterized by high density and promiscuity between semidomestic and wild animals. Two cases of CWD have been reported in Finland since March 2018, and three cases were reported in Sweden since March 2019 [4] (Figure 1).

Based on prevalence data from Norway and the United States, it is likely that CWD has been circulating in Europe for at least a decade. In response, the European Union (EU) set up an emergency 3-year plan (1 January 2018–31 December 2020) to reinforce the surveillance system (EU Regulation no. 2017/1972, 30 October 2017) in six member states or the countries of the European Free Trade Association with a sizable cervid population (Lithuania, Latvia, Finland, Estonia, Poland, and Sweden).

Given the presence of cervids in Italy and the growing concern about the spread of infection in Europe, the Italian Ministry of Health developed a monitoring plan to evaluate the risk of CWD. The plan is currently operating and targets adult cervids that are not fit for consumption.

According to the most recent (2010) estimate of the Italian deer population by the Italian Institute for Environmental Protection and Research, the autochthonous species are as follows: 17,697 fallow deer (*Dama dama*), 67,788 red deer, and 457,794 roe deer (*Capreolus capreolus*) [5].

**Figure 2:**
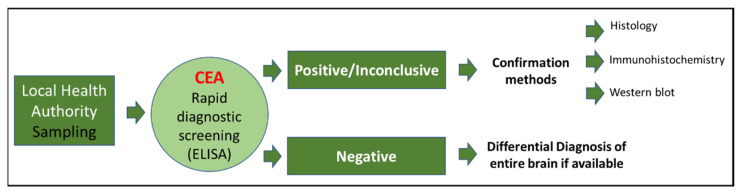
Flowchart for sample testing.

In the framework of surveillance, the sampling of the brainstem (obex), medial retropharyngeal lymph nodes, and, whenever possible, the brain and tonsils of wild and farmed cervids found dead or emaciated is carried out by a local health authority. The samples are sent to the local Istituto Zooprofilattico (Public Health Institute) and then to the CEA laboratory, which is a part of the Istituto Zooprofilattico Sperimentale del Piemonte, Liguria e Valle d’Aosta (IZSPLV), where the samples undergo rapid diagnostic screening tests. Positive or inconclusive samples undergo histological, immunohistochemical (IHC), and immunobiochemical (Western blot) confirmation (Figure 2).

CEA was designated a reference center by the Italian Ministry of Health (Decree of 3 August 1991). Starting 14 October 2015, the Center has provided technical-scientific support to the World Organization for Animal Health (OIE) as an OIE reference laboratory for BSE and scrapie. On 1 January 2019, CEA extended its activities as an EU reference laboratory for TSE. The CEA neuropathology laboratory carries out diagnostic services, research into neurological diseases in animals, and monitoring of prion diseases. In 2017, within the framework of the national CWD monitoring plan, the Center began conducting histopathological analysis of the brains of wild ruminants. The aim of the present study is to report the findings of the national CWD monitoring plan for a 3-year period (2017–2018–2019) and to describe the major patterns of neuropathological lesions and correlated pathogens.

## 2. Results

During the 3-year study period (1 January 2017 to 31 December 2019), the brain samples from 1758 cervids (570 in 2017, 609 in 2018, and 579 in 2019) underwent rapid testing, and all samples tested negative for CWD. The most numerous species were roe deer (1349), red deer (299), fallow deer (85), and a few captive reindeer (25). Most were wild (1675, 95.3%) and were categorized as fallen stock (1579, 89.8%, of which 1120 were roadkill and 459 had unknown causes of death) or culled because of sickness or because they displayed neurological signs. Animal age could be estimated in many species (1155, 66%), 76.1% of which were between 2 and 4 years old; males (when sex was reported) made up about half of the total population (862/1611, 53.1%).

As no CWD cases were detected out of the 1758 cervids, assuming a sensitivity of 1 and a 95% confidence level, the achievable design prevalence, i.e., the maximum prevalence compatible with zero cases in this high risk group, was very low, i.e., 0.17%.

**Figure 3:**
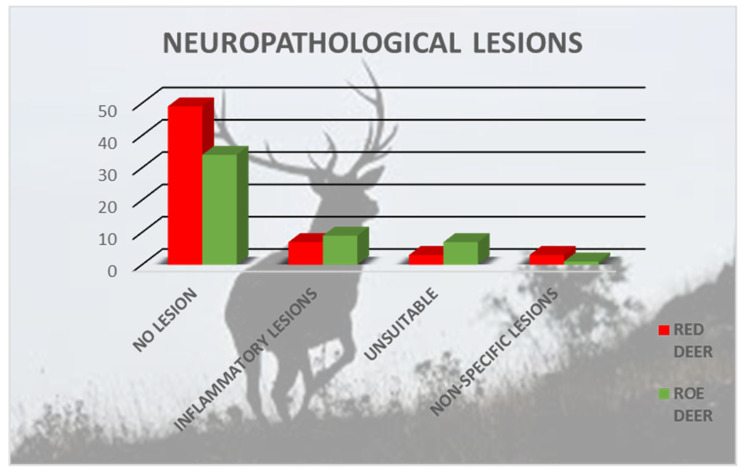
Neuropathological analysis of brain samples from 62 red deer and 51 roe deer.

A total of 113 brains (62 red deer and 51 roe deer) from the 1758 samples were analyzed for differential diagnosis. Most species that came from Northern Italy (102/113), were adults (77/113) and females of both species (67/113). Neuropathological lesions were observed in 20 animals: roe deer (n = 11) and red deer (n = 9) (Figure 3). Nine cervids were young (0–4 years), four were adults (4–8 years), and five were old (>8 years); nine were female and nine were male. Data for two individuals are missing. 

**Figure 4:**
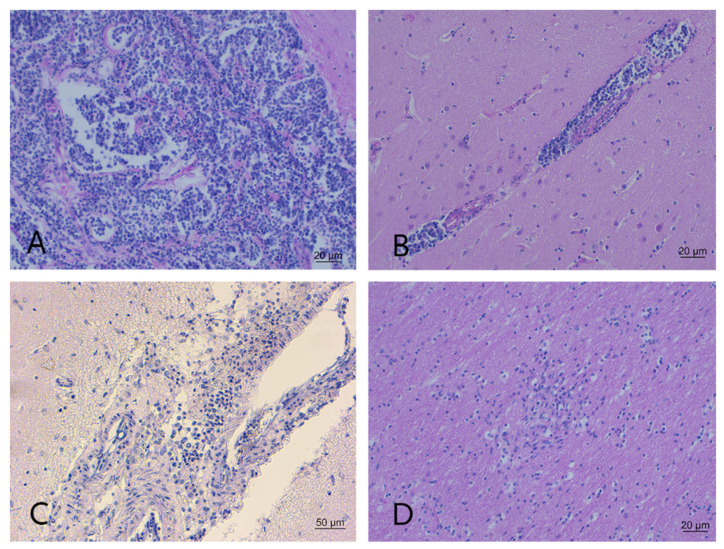
Neuropathological lesions. (**A**). Severe lymphoplasmacytic meningitis in the frontal cortex (red deer). HE; 20×. (**B**). Perivascular cuffing of mononuclear cells in the frontal cortex (red deer). HE; 20×. (**C**). Diffuse and severe suppurative meningoencephalitis characterized by infiltrate of polymorphonuclear cells (roe deer). HE; 10×. (**D**). Foci of gliosis in the parietal cortex (red deer). HE; 20×.

The brains from 10 animals were unsuitable for neuropathological examination because the individual was either in an advanced stage of autolysis or showed freezing artifacts; the brains from 83 individuals presented no lesions. In 15/20 animals (seven red deer and eight roe deer), histopathological analysis revealed mild-to-severe non-suppurative encephalitis or meningoencephalitis. The microscopic hallmarks were perivascular cuffs composed of single or multiple layers of mononuclear cells, vasculitis, and moderate gliosis depending on lesion severity. Severe lesions contained inflammatory cells in the surrounding parenchyma (Figure 4A,B,D).

Diffuse and severe suppurative meningoencephalitis characterized by an infiltrate of polymorphonuclear cells invading the cerebral parenchyma from the meninges was observed in the brain sample from one roe deer (Figure 4C). Perivascular cuffs, plexus choroiditis, and minor hemorrhage were also noted. Non-specific lesions in four animals were characterized by focal areas of hemorrhage and/or mild glial reactivity that made diagnostic interpretation difficult. 

Lesions in red deer were chiefly localized in a single brain area and differed among the individuals. The most frequent lesions in roe deer were characterized by inflammation of the choroid plexus and the meninges. 

Due to a flooding accident at the laboratory, many samples turned moldy, leaving intact only nine of the twenty paraffin-embedded samples with neuropathological lesions. Of these nine samples, three came from roe deer and five from red deer. Five came from young animals (0–4 years) and three from adults (4–8 years); five were from female and three from male individuals. Data for one individual are missing. Mild-to-severe non-suppurative encephalitis or meningoencephalitis was detected in eight samples, and non-specific lesions were detected in one sample. All nine samples tested negative for *Listeria* spp. and BoAstVCH13/Neuro S1 IHC and CvHV-1 biomolecular analyses. 

## 3. Discussion and Conclusions

During the 3-year study period, no cases of CWD were detected in this large sample of cervids collected via the Italian monitoring plan. The results indicate the absence of disease in the study area where the monitoring plan targets animals with known risk factors (age class compatible with the minimal hypothesized incubation period and testing of fallen stock or roadkill or culled because of sickness). This plan provided the material for analysis of neuropathological changes in wild ruminants and possible causes of death. 

Our findings show no correlation between neuropathological lesions and geographical areas of origin of the animals nor between lesions and sex or age. These results notwithstanding, our study underscores the advantage of brain sampling during the necropsy of wild animals as it can yield scientific information relevant for animal health. Brain examination helped to exclude the presence of CWD and provided a reference spectrum of neuropathological lesions that could be useful for differential diagnosis in Italian cervids of unknown clinical status and symptoms. Wild animals are often affected by emerging or re-emerging diseases of the central nervous system. With the exception for sporadic reports [6,7], the current literature on neuropathological lesions in wild animals is scarce, making it difficult to obtain a clear picture of the situation.

Based on the present literature, possible differential diagnoses according to the neuropathological lesions found in our samples, characterized by non-suppurative meningoencephalitis, could be ascribed to CvHV-1, BoAstVCH13/Neuro S1, *tick borne encephalitis virus* (TBEV), and *bovine viral diarrhea virus* (BVDV). Samples in this study were tested for CvHV-1 as it is a frequently reported disease in cervids, although with pathological localization mainly occurring at the ocular level [8,9]; BoAstVCH13/Neuro S1, instead, was studied as an emerging pathogen, described in ruminants in association with encephalitis [10]. The recent findings of Da Rold described animals affected by TBE with a moderate, multifocal encephalitis characterized by perivascular cuffs and neuropil infiltrates of lymphocytes and histiocytes in both grey and white matter similar to the lesions present in some of our samples [11]. In addition, minimal to mild non-suppurative meningoencephalitis referable to BVDV is relatively common in adult cervids [12]. However, it was not possible to test the samples in this work for TBEV or BVDV because the method of RNA extraction from paraffin-embedded samples with the AllPrep DNA/RNA FFPE Kit (QIAGEN) is under validation in our laboratories.

*Listeria* spp. neuropathological lesions are typically a mixture of non-suppurative and suppurative patterns [13,14]; therefore, they were included among the differential diagnoses in this study. Another etiologic agent attributable occasionally to suppurative brain injuries described in the literature was *Brucella* spp. [15]. Unfortunately, with respect to the one specimen with suppurative lesions, there were no tissues available that were either frozen or paraffin-embedded; thus, it could not be investigated.

A study of a larger number of paraffin-embedded and frozen tissue samples would, therefore, allow for a more comprehensive investigation into the neuropathological role of zoonotic pathogens in the cervid population.

The recent evolution of the epidemiology of CWD in Europe raises cause for concern about the risk of a spread of the disease and calls for effective management. Data from North America indicate that once CWD has penetrated an area, it becomes endemic and is difficult to eradicate [16,17]. Furthermore, the current uncertainty regarding its zoonotic potential needs to be settled with effective prophylactic measures today and in the future to prevent its introduction and spread.

## 4. Materials and Methods

Specific guidelines for CWD monitoring have been issued by the Italian Ministry of Health. In an effort to maximize the probability of detecting the disease, the monitoring plan covers all cervids over 18 months of age and those not fit for human consumption: Healthy hunted or slaughtered animals are excluded. The monitoring entails, in descending order of priority, the following risk categories of wild or captive cervids: fallen stock (dead animals or roadkill) and animals culled because of sickness or showing neurological symptoms attributable to TSE. To standardize data collection, a national sampling form was created: The analysis is based on the data reported on the form and the diagnostic outcome. A breakdown of the data is given by animal species, year of sampling, geographical area of sampling, risk category, and outcome (by tissue type). Statistical analysis was performed using Stata 16. The achievable design prevalence associated with the overall sample size of animals tested in this study was obtained by using Epitools (https://epitools.ausvet.com.au/herdsensfive, accessed on 21 March 2022).

Between 2017 and 2019, a total of 1758 samples were analyzed using Idexx Herd Check (Bovine Spongiform Encephalopathy Antigen Test Kit, EIA, One IDEXX Drive, Westbrook, ME, USA). The test was carried out according to the manufacturer’s instructions. Briefly, the homogenates were mixed with 30 μL of the working plate diluent solution (D1 and D2); 100 μL of the mixture was loaded onto the antigen-capture plate and shaken for 45 min at room temperature. After washing, the plate was incubated in 100 μL of conditioning buffer for 10 min. Abnormal PrP was detected using 100 μL of the kit-conjugated anti-PrP antibody, CC, or SRB-CC (45 min incubation); visualised with 100 μL TMB (15 min incubation); and its absorbance was read at 450 nm and 620 nm.

Animals testing negative and for which the entire brain was available underwent histopathological analysis for differential diagnosis. For neuropathological examination, the samples were fixed in 10% buffered formaldehyde solution. The coronal sections of selected brain regions (telencephalon, diencephalon, mesencephalon, pons, cerebellum, and obex) were stained with hematoxylin and eosin (H&E). Samples with neuropathological lesions were submitted to IHC for the detection of *Listeria* spp. and neurotropic bovine astrovirus (BoAstVCH13/Neuro S1) and to biomolecular analysis for *cervid herpes virus* (CvHV-1). IHC for *Listeria* spp. was performed using polyclonal rabbit antibody, diluted at 1:250 (Virostat, Portland, ME, USA), and applied overnight at 4 °C (Vectastain ABC kit, peroxidase rabbit IgG, Vector Laboratories, Inc., Burlingame, CA, USA) [18,19]. The positive and negative controls were from ovine tissues previously tested by IHC and culture. As described in the procedure validated by Buojon et al., 2016, the IHC screening test for astrovirus was performed using polyclonal rabbit antibody BoAstVCH13/Neuro S1 (kindly provided by the Division of Neurological Sciences, Vetsuisse Faculty, University of Bern, Switzerland), diluted at 1:100, and applied overnight at 4 °C (Vectastain ABC kit, peroxidase rabbit IgG, Vector Laboratories, Inc., Burlingame, CA, USA) [20]. The positive and negative controls for IHC analysis were from bovine tissues previously tested and classified by RT-PCR.

Due to the lack of frozen tissue, the purification of genomic DNA from formalin-fixed, paraffin-embedded (FFPE) tissue sections was performed using the AllPrep DNA/RNA FFPE Kit (QIAGEN, Hilden, Germany) to detect CvHV-1. Real-time PCR was performed to detect viral DNA of BoHV-1 strains using a pair of sequence-specific primers of glycoprotein B (gB), gB-Forward: 5′-TGT-GGA-CCT-AAA-CCT-CAC-GGT-3′ (position 57499–57519 GenBank^®^, accession AJ004801) and gB-Reverse: 5′-GTA-GTC-GAG-CAG-ACC-CGT-GTC-3′ (position 57595–57575 GenBank^®^, accession AJ004801), for the amplification of DNA and a labelled probe, 5′-FAM-AGG-ACC-GCGAGT-TCT-TGC-CGC-TAMRA-3′ (position 57525–57545 GenBank^®^, accession AJ004801), was used for the detection of amplified products. The complete real-time PCR test procedure is described in the OIE Terrestrial Manual, Chapter 3.4.11 [21].

## Data Availability

Not applicable.
